# A painless glomus tumor: a case report

**DOI:** 10.1186/s13256-018-1837-2

**Published:** 2018-10-18

**Authors:** Ouiame EL Jouari, Salim Gallouj, Sara Elloudi, Ghita Senhaji, Mouna Rimani, Fatima Zahra Mernissi

**Affiliations:** 1 Department of Dermatology University Hospital Hassan II, Fez, Morocco; 2 Faculty of Medicine, Tangier, Morocco; 3 Hassan Center of Anatomopathology, Rabat, Morocco

**Keywords:** Glomus tumor, Painless, Dermoscopy, Histology, Surgery

## Abstract

**Background:**

Glomus tumor is a benign and vascular hamartoma that originates from the neuromyoarterial cells of the normal glomus apparatus in the reticular dermis. The etiology of glomus tumors is unknown. It usually presents as a small, slightly raised, bluish or pinkish-red, painful nodule of the fingertips and the pulp. we report an atypical case of a patient of painless glomus tumor.

**Case presentation:**

Our patient, a 60-year-old Moroccan man, had a 2.5 cm purplish painless soft tumor, covered with melliciric and hemorrhagic crusts, involving the first phalanx of his right index finger. This tumor was compressing his nail plate. No bony lesions were identified by radiographic studies, but magnetic resonance imaging was suggestive of glomus tumor. Surgical excision was performed with directed healing.

**Conclusions:**

The diagnosis of a glomus tumor is an eventuality even in the absence of pain.

## Background

Glomus tumor is a benign and vascular hamartoma that originates from the neuromyoarterial cells of the normal glomus apparatus in the reticular dermis [1]. It accounts for 1–5% of soft tissue tumors of the hand [2]. This tumor typically presents with cold hypersensitivity, pain, tenderness, and sometimes nail deformities or nail discoloration [3]. Although the precise cause of glomus tumors is unknown [4]. We report an atypical case of a patient with painless glomus tumor.

## Case presentation

**Fig. 1:**
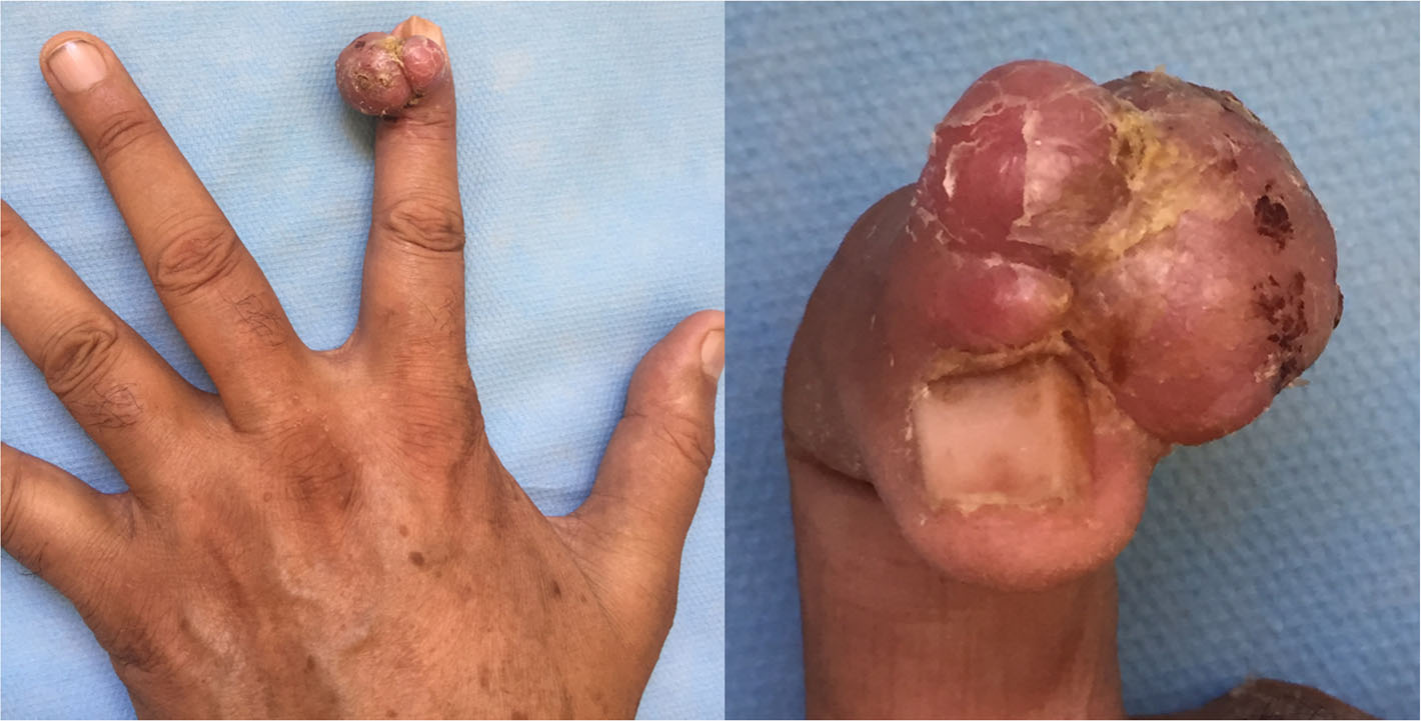
Purplish painless soft tumor, covered with yellowish hemorrhagic crusts, involving the index finger and deforming the nail

**Fig. 2:**
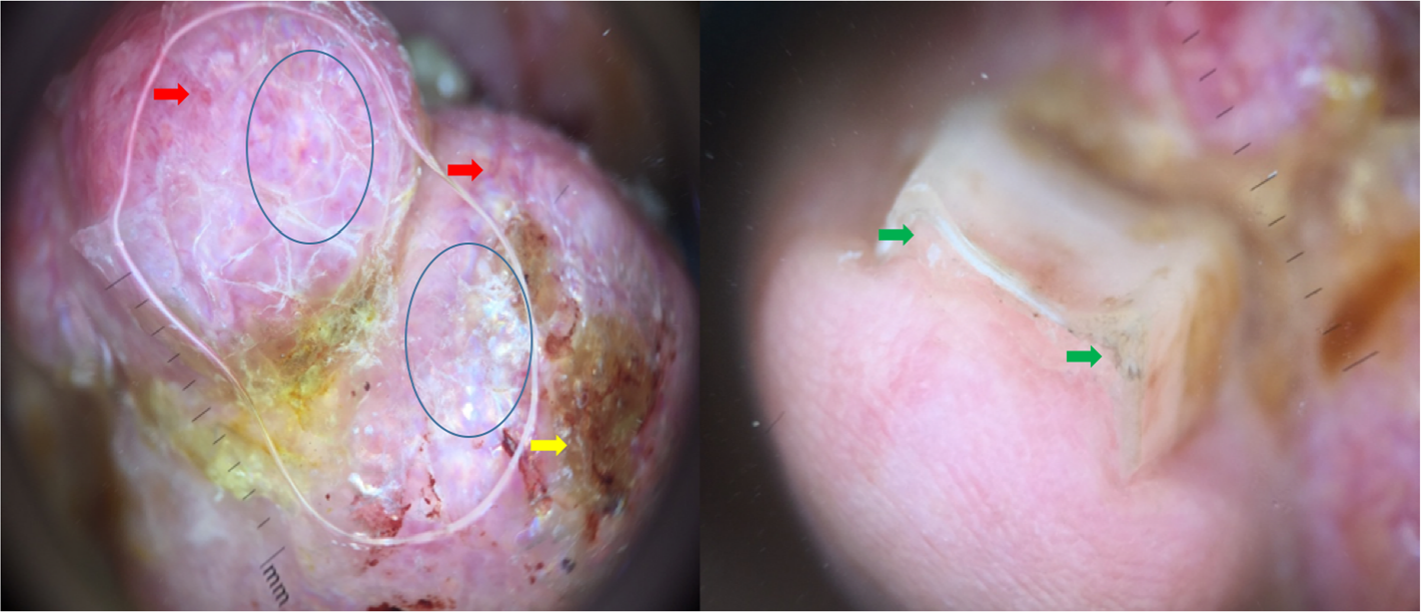
Polymorphic vessels (*red arrows*), rainbow pattern (*blue circles*) with yellowish, hemorrhagic crusts (*yellow arrows*), and deformation of the nail (*green arrows*)

**Fig. 3:**
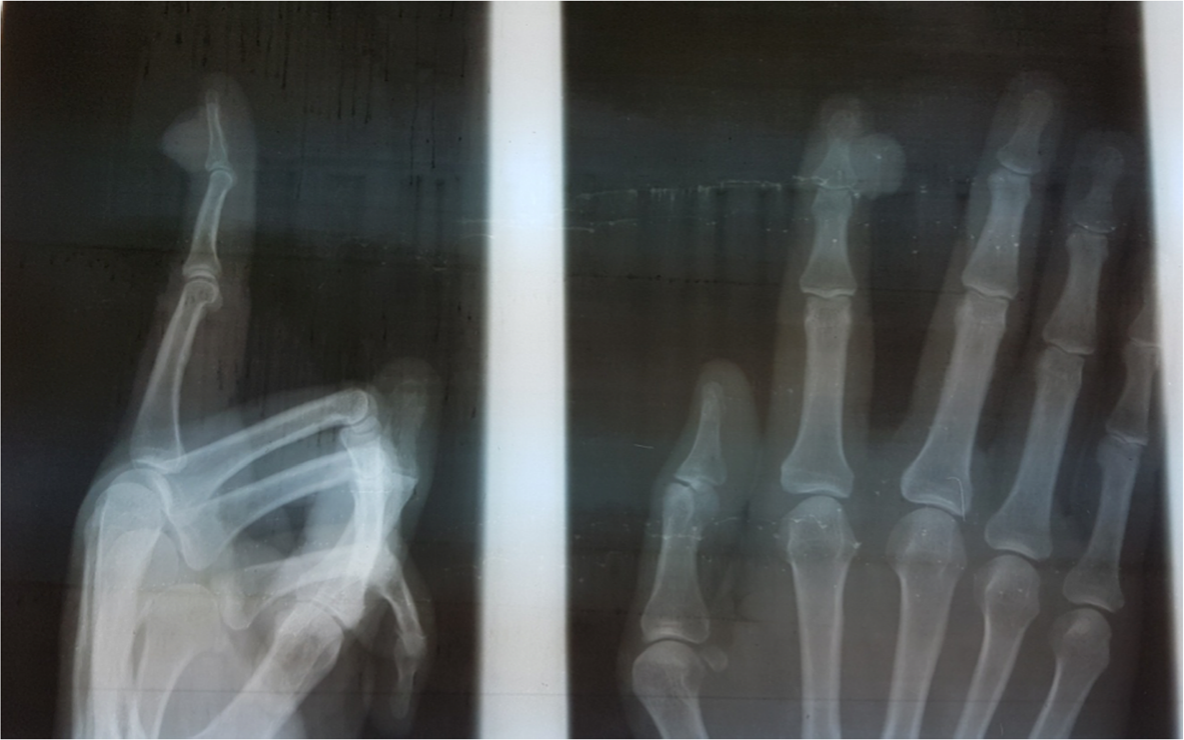
Radiography of the hand, face and profile: no bony lesions

**Fig. 4:**
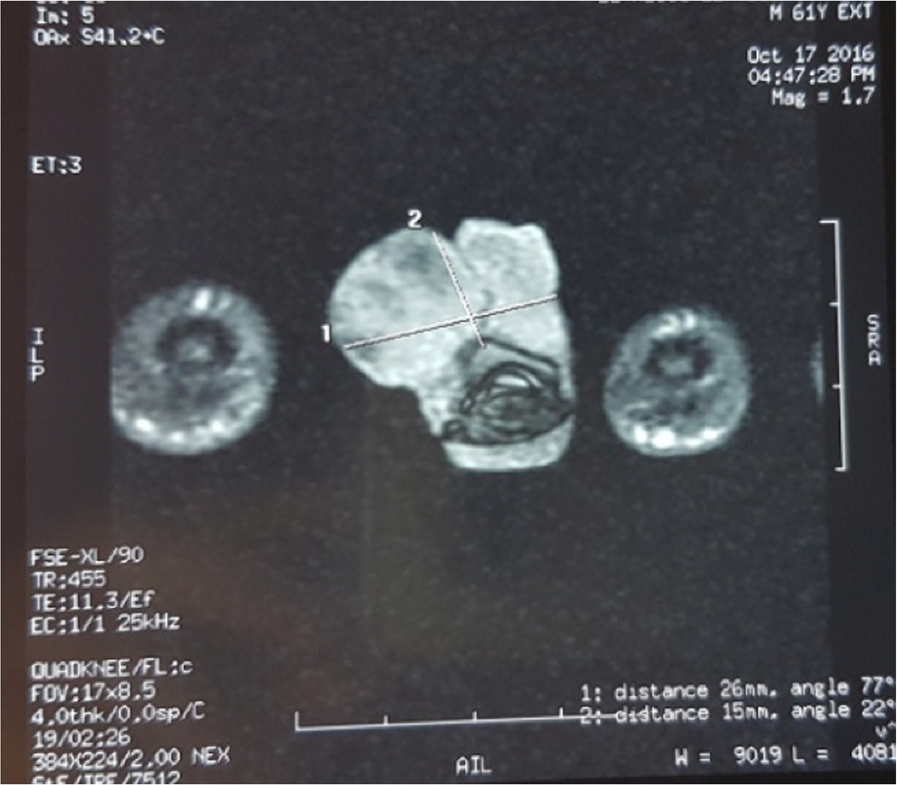
Magnetic resonance imaging: 26 × 16 mm low tissue mass signal intensity on T1, marked hyperintensity on T2, and enhancement on T1 after gadolinium injection.

A 60-year-old Moroccan man, without a personal history of diabetes or chronic disease, nor any special chirurgical or psychosocial background or toxic habits, and with a familial history of diabetes. He presented with a 3-year history of a progressively asymptomatic nodule of his right index finger. The tumor was voluminous, which motivated the patient to consult in our department. The clinical examination revealed a 2.5 cm purplish painless soft tumor, covered with yellowish and hemorrhagic crusts, involving the first phalanx of the right index finger. This tumor was compressing the nail plate (Fig. 1). Our patient did not report any intense pain, cold sensitivity or severe tenderness to palpation of the tumor of his right index finger, and no previous trauma history. A neurologic examination showed no signs of paresthesia or hypoesthesia, and muscular and neurological function was preserved. The dermoscopic examination had revealed polymorphic vessels, in a rainbow pattern with melliciric and hemorrhagic crusts (Fig. 2). A general examination showed no other abnormality. The differential diagnosis included angifibroma, pyogenic, granuloma-like Kaposi sarcoma, epidermized pyogenic granuloma, superficial acral fibromyxoma and glomus tumor. No bony lesions were identified on radiographic studies (Fig. 3) and magnetic resonance imaging (MRI) was suggestive of glomus tumor by individualizing a 26 × 16 mm low tissue mass signal intensity on T1, marked hyperintensity on T2, and enhancement on T1 after gadolinium injection (Fig. 4).

**Fig. 5:**
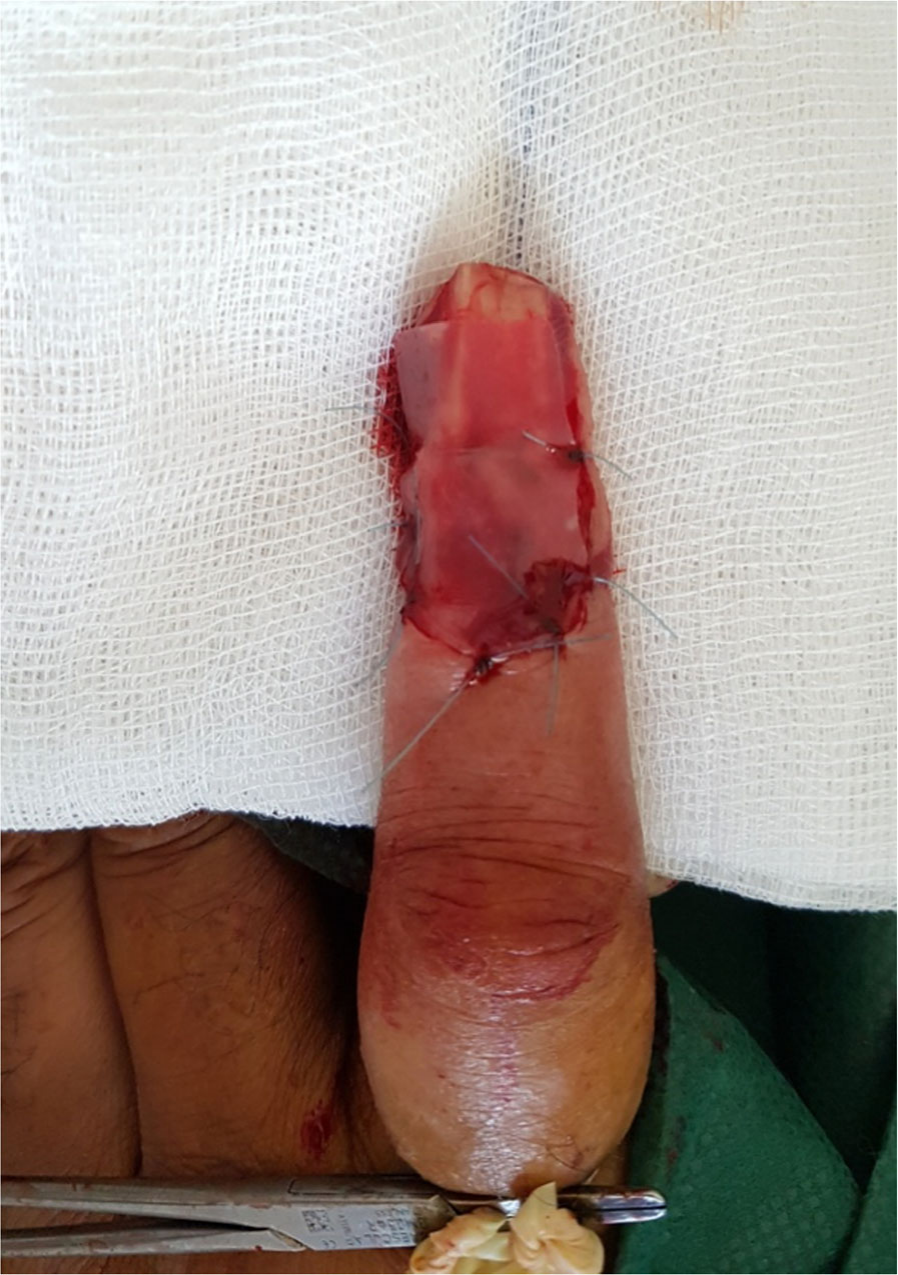
Surgical excision of glomus tumor

**Fig. 6:**
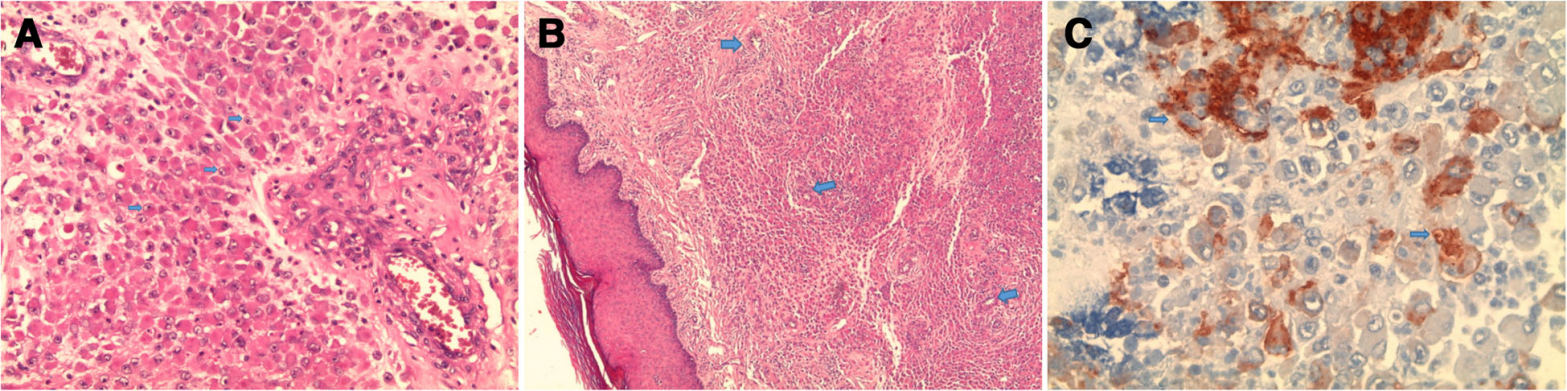
(**a**) Hematoxylin-eosin-saffron stain G × 200 - > Proliferation of ovoid cells (*blue arrows*). (**b**) Hematoxylin-eosin-saffron stain G × 50: Dermal proliferation getting organized around vascular clefts (*blue arrows*). (**c**) Immunohistochemistry G × 400: Antibody anti-acute myeloid leukemia (*blue arrows*)

Surgical excision was performed. The approach was direct, respecting the principles of cutaneous incisions and avoiding nerve fiber pathways. The mass was well circumscribed and removed (Fig. 5). Histopathologic examination with hematoxylin-eosin stain, demonstrated round to ovoid cells, lacking nuclear atypia and featuring scant, eosinophilic cytoplasm (Fig. 6a). The cell clusters were traversed by narrow vascular clefts lined with regular flattened endothelial cells (Fig. 6b). Mitotic activity was absent. Immunohistochemistry with anti-smooth muscle antibody supported the diagnosis of glomus tumor by demonstrating tumoral smooth muscle actin (Fig. 6c). At follow-up visits, no further radiological investigations were requested and no recurrence was noted. There was complete healing of the finger within 6 months and the nail regained its normal appearance in 10 months.

## Discussion

Glomus tumor is known as a benign and vascular hamartoma containing all the neuromyoarterial cells of the normal glomus apparatus [1]. These glomus bodies are contractile tissue and are primarily responsible for local temperature and blood pressure modulation, and they accomplish this by controlling blood flow through microvasculature [4]. The etiology of glomus tumors is unknown and it may be related to sex, age, trauma, or inheritance. Some authors have proposed that a weakness in the structure of a glomus body could lead to reactive hypertrophy after trauma. A familial variant of glomus tumor had been linked to chromosome 1p21–22 and involved truncating mutations in the glomulin gene, which encoded a 68-kDa protein with unknown function [2]. Young adults, mostly women, are primarily affected [5]. The tumor most commonly arises in the fingertips and the pulp [1]. It usually presents as a small, slightly raised, bluish or pinkish-red, painful nodule, and when subungual in location, can elevate, deform and discolor the nail [2]. Glomus tumor manifests with three symptoms: hypersensitivity to cold, heightened pinprick sensitivity, and paroxysmal pain [4]. To the best of our knowledge, we describe the first case of a patient with painless glomus tumor. In our case, the second particularity was that the tumor was very voluminous inducing deformation of the nail. The diagnosis of glomus tumor should involve positive results on tests: Love’s pin test, a cold sensitivity test, and Hildreth’s test [5]. Love’s pin test utilizes the head of a pin pressed against the site of the pain to identify the focal point. For Hildreth’s test, the patient’s lesion must be first stimulated to provoke severe pain. After that, a tourniquet is applied, and Love’s pin test is repeated; the absence of pain from the pin after applying the tourniquet indicates a positive result for Hildreth’s test. A positive result on the cold sensitivity test manifests as an increase in pain due to the cold. The mechanism for this may depend on the vasodilation of the Souquet–Hoyer arteriovenous channels, which dilate in response to cold to prevent excessive digit heat loss [6].

Radiographs can show cortical thinning or erosive changes in the adjacent bone in some of the cases [2]. Imaging studies such as ultrasound and MRI can be valuable tools for ruling out possibilities, visualizing, and diagnosing glomus tumors [4].

Ultrasonography is capable of demonstrating the size, site, and shape of the tumor, but is frequently influenced by the surgeon’s experience [2, 3].

Typical characteristics of a glomus tumor on MRI are low signal intensity on T1-weighted images, marked hyperintensity on T2-weighted images, and enhancement on T1-weighted images after gadolinium injection [1].

Here, despite the fact that the tumor was located in a preferential zone for glomus tumor, MRI was necessary for the diagnosis because of the absence of the painful character that is pathognomonic of these tumors.

Barre and Masson described the histology of glomus tumor for the first time [3].

Histology reveals a variable composition of glomus cells, blood vessels, and smooth muscles. Based on this, glomus tumors are categorized into three types: glomangiomas with an abundance of vessels; solid glomus tumor, chiefly composed of glomus cells; and glomangiomyomas showing a predominance of smooth muscles [2].

Complete surgical excision is the curative treatment of choice for glomus tumor [1]. Incomplete excision is considered as the main cause of recurrence [2].

We aimed to emphasize, by reporting this case, the importance of inclusion of glomus tumor among the possibilities for differential diagnosis of digital nodules, even if painless.

## Conclusions

Glomus tumors are rare tumors with a classic clinical presentation and typical symptoms of long-term pain and sensitivity to touch. We report the case of a patient with an unusual painless glomus tumor.
